# Activity of Oritavancin against Gram-Positive Pathogens Causing Bloodstream Infections in the United States over 10 Years: Focus on Drug-Resistant Enterococcal Subsets (2010–2019)

**DOI:** 10.1128/AAC.01667-21

**Published:** 2022-02-15

**Authors:** Cecilia G. Carvalhaes, Helio S. Sader, Jennifer M. Streit, Mariana Castanheira, Rodrigo E. Mendes

**Affiliations:** a JMI Laboratories, North Liberty, Iowa, USA

**Keywords:** lipoglycopeptides, *E. faecium*, VRE, VanA, VanB, vancomycin resistance, daptomycin resistance

## Abstract

Oritavancin displayed potent and stable activity (MIC_90_ range of 0.06 to 0.5 mg/L) over a 10-year period (2010 to 2019) against Gram-positive pathogens that cause bloodstream infections (BSI), including methicillin-resistant Staphylococcus aureus (MRSA) and resistant subsets of *Enterococcus* spp. Daptomycin and linezolid were also active against methicillin-resistant S. aureus and vancomycin-resistant *Enterococcus* (VRE). Only oritavancin and linezolid remained active against Enterococcus faecium isolates displaying an elevated daptomycin MIC (i.e., 2 to 4 mg/L). Proportions of methicillin-resistant S. aureus and vancomycin-resistant *Enterococcus* within the respective S. aureus and enterococcal populations decreased over this period.

## INTRODUCTION

Bloodstream infections (BSI) are a major cause of morbidity and mortality among healthcare- and community-associated infections. In this scenario, the emergence and global spread of multidrug-resistant organisms, including methicillin-resistant Staphylococcus aureus (MRSA), vancomycin-resistant *Enterococcus* spp. (VRE), and carbapenem-resistant *Enterobacterales* imposes a serious challenge for treating BSI caused by these pathogens (1–3).

Oritavancin is a lipoglycopeptide agent with a prolonged half-life and concentration-dependent bactericidal activity against clinically relevant Gram-positive pathogens (4). Previous studies have demonstrated that, in addition to acting against methicillin-susceptible S. aureus (MSSA), streptococci, and vancomycin-susceptible enterococci isolates, oritavancin shows potent activity against resistant isolates, such as MRSA and VRE (5–8).

The efficacy and safety of a single 1,200-mg dose of oritavancin over a 3-h infusion (Orbactiv) was demonstrated previously in clinical trials (SOLO I and SOLO II studies) for treating patients with acute bacterial skin and skin structure infection (ABSSSI) (4, 9, 10). More recently, the same oritavancin dose (1,200 mg) with a shorter infusion duration time (1 h; Kimyrsa) was approved by the US FDA (11, 12), providing additional flexibility in treating patients with moderate or severe ABSSSI. This study evaluated the activity of oritavancin against a collection of Gram-positive pathogens and resistant subsets causing BSI in US medical during a 10-year (2010 to 2019) period. This study expands on a previous analysis of oritavancin activity against US and European *Enterococcus* species isolates during 2011 to 2014 (5).

Throughout 2010 to 2019, 15,403 Gram-positive bacterial pathogens causing BSI (1 per patient episode) were collected from 36 medical centers across all 9 US Census Divisions. Bacterial confirmatory identification was performed by JMI Laboratories (North Liberty, IA) using matrix-assisted light desorption ionization-time of flight mass spectrometry (MALDI-TOF MS) and standard microbiology methods, such as bile solubility and susceptibility to optochin (see the Table 1 footnotes for a list of all isolates included in the study). Susceptibility testing was performed by broth microdilution following CLSI methods (13), using either dry-form (2010 to 2014) (Thermo Fisher; Bedford, MA) or frozen-form (2015 to 2019) panels (JMI Laboratories). MIC interpretations were based on CLSI criteria (14).

**TABLE 1:** Oritavancin activity and occurrence of resistance phenotypes among Gram-positive isolates that cause BSI in US medical centers (2010 to 2019)^*a*^

Organism or organism group (no. of isolates)	Oritavancin MIC_50_/MIC_90_ (mg/L) and % susceptible (CLSI^*b*^) per yr group												
	2010–2011		2012–2013		2014–2015		2016–2017		2018–2019		All yrs		MIC range (mg/L)
	MIC_50_/MIC_90_	%S	MIC_50_/MIC_90_	%S	MIC_50_/MIC_90_	%S	MIC_50_/MIC_90_	%S	MIC_50_/MIC_90_	%S	MIC_50_/MIC_90_	%S	
S. aureus (7,498)	0.03/0.06	99.5	0.03/0.06	100.0	0.015/0.06	99.9	0.015/0.06	99.9	0.03/0.06	99.9	0.03/0.06	99.8	≤0.008–0.25
MRSA (3,226)	0.03/0.06	99.8	0.03/0.06	100.0	0.015/0.06	99.8	0.015/0.06	99.7	0.03/0.06	100.0	0.03/0.06	99.8	≤0.008–0.25
MSSA (4,272)	0.03/0.06	99.2	0.03/0.06	100.0	0.015/0.06	100.0	0.015/0.06	100.0	0.03/0.06	99.9	0.03/0.06	99.8	≤0.008–0.25
CoNS^*c*^ (1,872)	0.03/0.06	99.4	0.03/0.06	100.0	0.03/0.06	99.8	0.03/0.12	98.5	0.06/0.12	95.3	0.03/0.12	98.4	≤0.008–1
MRCoNS (1,163)	0.03/0.06	99.5	0.03/0.06	100.0	0.03/0.06	99.6	0.03/0.12	97.5	0.06/0.12	94.0	0.03/0.12	97.9	≤0.008–0.5
MSCoNS (709)	0.015/0.06	99.2	0.015/0.06	100.0	0.03/0.06	100.0	0.03/0.06	100.0	0.03/0.06	97.0	0.03/0.06	99.2	≤0.008–1
VGS^*d*^ (921)	0.015/0.12	100.0	≤0.008/0.06	100.0	0.015/0.06	100.0	0.015/0.25	99.3	0.015/0.25	93.8	0.015/0.12	98.7	≤0.008–0.5
BHS^*e*^ (1,394)	0.03/0.12	100.0	0.03/0.12	99.1	0.03/0.12	98.6	0.06/0.25	97.1	0.06/0.25	98.2	0.03/0.25	98.5	≤0.008–1
													
*Enterococcus* spp. (2,895)	0.015/0.06	97.0	0.015/0.03	98.0	0.015/0.06	98.1	0.015/0.03	99.4	0.015/0.06	98.1	0.015/0.06	97.9	≤0.008–0.5
E. faecalis (1,709)	0.015/0.06	96.2	0.015/0.03	97.4	0.015/0.03	98.3	0.015/0.03	99.1	0.015/0.03	97.8	0.015/0.06	97.5	≤0.008–0.5
Vancomycin-NS (≥8 mg/L) (62)	0.25/0.5	33.3	0.25/-	28.6	0.12/-	50.0	0.12/0.25	75.0	0.25/-	12.5	0.25/0.5	40.3	0.008–0.5
VanA phenotype (53)	0.25/0.5	25.0	0.25/-	16.7	0.25/-	20.0	0.12/0.25	75.0	0.25/-	12.5	0.25/0.5	32.7	0.015–0.5
VanB phenotype (9)	0.015/-	100.0	0.015	100.0	0.015/-	100.0	-	-	-	-	0.015/-	100.0	0.008–0.25
Daptomycin-NS (≥4 mg/L) (8)	0.03/-	100.0	0.03/-	100.0	0.015/-	100.0	0.03	100.0	-	-	0.03/-	100.0	0.008–0.06
Linezolid-NS (≥4 mg/L) (2)	≤0.008	100.0	-	-	-	-	-	-	0.008	100.0	≤0.008/-	100.0	≤0.008
E. faecium (1,082)	0.03/0.12	98.0	0.03/0.12	98.6	0.015/0.12	97.6	0.015/0.06	100.0	0.015/0.06	98.4	0.03/0.06	98.4	≤0.008–0.5
Vancomycin-NS (≥8 mg/L) (784)	0.03/0.12	97.5	0.03/0.12	98.2	0.03/0.12	96.5	0.03/0.06	100.0	0.03/0.06	97.4	0.03/0.12	97.8	≤0.008–0.5
VanA phenotype (755)	0.03/0.12	97.5	0.03/0.12	98.2	0.03/0.12	96.3	0.03/0.06	100.0	0.03/0.06	97.4	0.03/0.12	97.0.8	≤0.008–0.5
VanB phenotype (29)	≤0.008/≤0.008	100.0	≤0.008/-	100.0	≤0.008/-	100.0	-	-	≤0.008/-	100.0	≤0.008/0.03	100.0	≤0.008–0.06
Daptomycin-R (≥8 mg/L) (9)	≤0.008/-	100.0	-	-	≤0.008/-	100.0	0.015/-	100.0	0.06/-	66.7	0.015/-	88.9	≤0.008–0.25
Daptomycin MIC, 2–4 mg/L (540)	0.03/0.12	97.2	0.03/0.12	98.9	0.03/0.12	97.5	0.015/0.06	100.0	0.015/0.06	96.6	0.03/0.12	97.8	≤0.008–0.5
Linezolid-NS (≥4 mg/L) (13)	≤0.008/-	100.0	0.03	100.0	0.12	100.0	0.015/-	100.0	≤0.008/-	100.0	0.015/0.06	100.0	≤0.008–0.12
Ampicillin-R (≥16 mg/L) (945)	0.03/0.12	97.9	0.03/0.12	98.5	0.03/0.12	97.2	0.015/0.06	100.0	0.03/0.06	97.9	0.03/0.12	98.2	≤0.008–0.5
Other *Enterococcus* spp.^*f*^ (104)	≤0.008/0.015	100.0	≤0.008	100.0	≤0.008/0.03	100.0	≤0.008/0.015	100.0	≤0.008/0.015	100.0	≤0.008/0.015	100.0	≤0.008–0.06

a MRSA, methicillin-resistant Staphylococcus aureus; MSSA, methicillin-susceptible S. aureus; MRCoNS, methicillin-resistant coagulase-negative Staphylococcus; MSCoNS, methicillin-susceptible coagulase-negative Staphylococcus; VGS, Viridans group streptococci; BHS, beta-hemolytic streptococci; R, resistant; NS, nonsusceptible.

b Using CLSI (14) breakpoints. The oritavancin-susceptible breakpoint for S. aureus was applied to CoNS. The oritavancin-susceptible breakpoint for vancomycin-susceptible E. faecalis was applied to all *Enterococcus* species isolates.

c Organisms included: Staphylococcus arlettae (1), *S. auricularis* (14), *S. capitis* (115), *S. caprae* (10), *S. cohnii* (10), *S. condimenti* (1), S. epidermidis (1,133), *S. haemolyticus* (80), S. hominis (220), S. intermedius (1), *S. lugdunensis* (64), *S. pasteuri* (2), *S. pettenkoferi* (21), S. pseudintermedius (3), *S. saprophyticus* (10), *S. schleiferi* (4), S. sciuri (1), *S. simulans* (15), *S. warneri* (36), and Staphylococcus spp. (131).

d Organisms included: Streptococcus alactolyticus (2), *S. anginosus* group (89), *S. australis* (6), S. bovis group (28), *S. constellatus* (11), *S. cristatus* (7), S. equinus (1), S. gallolyticus (41), S. gordonii (23), *S. infantarius* (1), *S. infantis* (5), S. intermedius (12), *S. lutetiensis* (10), S. mitis group (393), S. mutans (12), *S. parasanguinis* (61), *S. salivarius* group (75), S. sanguinis (52), *S. sinensis* (1), *S. vestibularis* (9), and viridans group Streptococcus spp. (82).

e Organisms included: Streptococcus agalactiae (747), S. canis (7), S. dysgalactiae (143), S. equi (1), and S. pyogenes (496).

f Organisms included: Enterococcus avium (15), *E. casseliflavus* (23), *E. durans* (9), *E. gallinarum* (33), *E. hirae* (8), *E. raffinosus* (12), and *Enterococcus* spp. (4).

S. aureus (48.7%) alone comprised almost half of Gram-positive pathogens. The proportion of methicillin resistance among S. aureus varied from 46.6% to 42.3%, and these rates appeared to trend lower over time (Table 2). This trend was also noted in other regional and national surveillance programs during the 2000s, likely as a result of an increasing emphasis on hospital infection prevention, stewardship programs, and activities directed toward healthcare quality improvement (2, 15–18). Similarly, a progressive decrease in the rate of methicillin resistance among coagulase-negative Staphylococcus (MRCoNS) BSI was noted. Oritavancin MIC_50_ values against S. aureus ranged from 0.015 to 0.03 mg/L, and MIC_90_ values were 0.06 mg/L irrespective of methicillin susceptibility (Table 1). Oritavancin susceptibility rates of >99.5% were observed during this period for S. aureus (99.5% in 2010 to 2011). Oritavancin displayed MIC_50_ values of 0.03 to 0.06 mg/L against CoNS. Oritavancin inhibited 98.4% to 100.0% of CoNS isolates at ≤0.12 mg/L, except during 2018 to 2019 (95.3% susceptible). Oritavancin susceptibility remained stable (>95%) across the years against MSSA, MRSA, methicillin-susceptible CoNS, and MRCoNS, and was comparable to vancomycin, daptomycin, and linezolid (see Table S1 in the supplemental material).

**TABLE 2:** Evolution of resistance phenotypes of Staphylococcus spp. *and Enterococcus* species isolates from BSI in US medical centers^*a*^

	Rates of resistance (%) per study period^*b*^					
Resistance phenotype	2010–2011	2012–2013	2014–2015	2016–2017	2018–2019	All yrs
MRSA	46.6	40.1	43.7	40.4	42.3	43.0
MRCoNS	64.3	62.9	64.7	61.4	58.3	62.1
E. faecalis						
VRE (≥8 mg/L)	4.5	3.6	3.4	3.7	2.2	3.6
VanA phenotype	81.5	85.7	62.5	100.0	100.0	85.5
VanB phenotype	18.5	14.3	37.5	0.0	0.0	14.5
E. faecium						
VRE (≥8 mg/L)	79.6	77.4	67.7	66.5	62.8	72.5
VanA phenotype	96.6	98.2	95.5	96.7	93.9	96.3
VanB phenotype	3.4	1.8	4.5	3.3	6.1	3.7
Daptomycin-R (≥8 mg/L)	0.5	0.0	1.2	1.1	1.6	0.8
Daptomycin MIC, 2–4 mg/L	62.4	60.3	48.2	33.5	31.7	49.9
Linezolid-NS (≥4 mg/L)	1.7	0.7	0.6	1.1	1.1	1.2
Ampicillin-R (≥16 mg/L)	92.6	90.4	87.2	81.3	79.2	87.3

a MRSA, methicillin-resistant Staphylococcus aureus; MRCoNS, methicillin-resistant coagulase-negative Staphylococcus; VRE, vancomycin-resistant *Enterococcus;* R, resistant; NS, nonsusceptible.

b Using CLSI (14) breakpoints.

*Enterococcus* spp. comprised 18.8% (2,895 out of 15,403) of Gram-positive pathogens causing BSI, where E. faecalis was the most common species (59.0%), followed by E. faecium (37.4%). VRE rates within E. faecalis decreased over time, from 4.5% (2010 to 2011) to 2.2% (2018 to 2019). Likewise, vancomycin resistance (from 79.6% to 62.8%) and ampicillin resistance (from 92.6% to 79.2%) decreased in E. faecium (Table 2). Ampicillin resistance and VRE phenotypes were displayed by most E. faecium (87.3% were ampicillin resistant and 72.5% were VRE), whereas only 3.6% of E. faecalis were resistant to vancomycin and none were resistant to ampicillin (Table S2). The decline in VRE as a proportion of total enterococcal infections may be due to the same reasons as described above for MRSA (2, 19). The past increase in VRE in the US was mostly due to the expansion of E. faecium clonal complex 17 (20). The increase in ampicillin and vancomycin susceptibility may indicate a change in the epidemiology of E. faecium causing BSI in the United States. However, this epidemiology information was not captured for this large collection.

Oritavancin activity against E. faecalis and E. faecium was stable between 2010 and 2019. Consistent MIC_50_ values of 0.015 mg/L and MIC_90_ values of 0.03 to 0.06 mg/L were observed in all years against E. faecalis. Oritavancin inhibited 96.2% (in 2010 to 2011) to 99.1% (in 2016 to 2017) of E. faecalis at ≤0.12 mg/L (Table 1). E. faecium displayed MIC_50_/MIC_90_ values of 0.03/0.12 mg/L during the 2010–2011 and 2012–2013 periods, whereas MIC_50_/MIC_90_ values of 0.015/0.06 mg/L were seen during the 2016–2017 and 2018–2019 periods. Oritavancin susceptibility rates remained stable against E. faecium across all time periods (97.6% to 98.6%). Many antimicrobials showed activity (>95%) against E. faecalis, such as ampicillin, daptomycin, linezolid, vancomycin, and oritavancin, while only daptomycin, linezolid, and oritavancin remained active against E. faecium (Table S2).

Oritavancin inhibited 97.7% of VanA and 100% of VanB E. faecium at ≤0.12 mg/L. In contrast, only 32.7% of E. faecalis isolates displaying the VanA phenotype were inhibited by oritavancin at ≤0.12 mg/L. VRE E. faecalis showed oritavancin MIC_50_ values ranging from 0.12 to 0.25 mg/L, whereas MIC_50_ values ranged from 0.015 to 0.03 mg/L against VRE E. faecium. The greater activity of oritavancin against E. faecium compared to E. faecalis is not well understood. Expression of *vanZ* or changes in LiaS sensor kinase were reported as possible explanations (21).

Almost half (49.9%) of E. faecium displayed daptomycin MICs of 2 to 4 mg/L, and 9 (0.8%) isolates were resistant (MIC, ≥8 mg/L; Table 1). The rates of E. faecium displaying elevated daptomycin MICs showed a progressive decrease over the study years, ranging from 62.4% (in 2010 to 2011) to 31.7% (in 2018 to 2019) (Table 2). Oritavancin activity against E. faecium with elevated MIC values against daptomycin (2 to 4 mg/L) remained stable throughout the study (MIC_50_/MIC_90_, 0.015 to 0.03/0.06 to 0.12 mg/L). Oritavancin inhibited >97% of VRE, linezolid-nonsusceptible, and E. faecium displaying elevated daptomycin MICs (2 to 4 mg/L) at ≤0.12 mg/L. Recent pharmacokinetic (PK) analysis provide evidence that multiple oritavancin doses may be beneficial in treating severe infections and can achieve serum concentrations above the E. faecalis susceptibility breakpoint of 0.12 mg/L, for over 4 weeks (22, 23). However, clinical studies are needed to evaluate the relationship between PK and clinical outcomes with oritavancin treatment. Another study conducted by Belley and colleagues on the pharmacodynamic activity of oritavancin against daptomycin-nonsusceptible VRE E. faecium suggested that a multiple-dose strategy with oritavancin may be effective against daptomycin-nonsusceptible vancomycin-resistant E. faecium (24).

Viridans group Streptococcus isolates displayed oritavancin MIC_50_/MIC_90_ values of ≤0.008 to 0.015/0.06 to 0.25 mg/L and susceptibility rates of 93.8% to 100.0% over the study period, while beta-hemolytic Streptococcus showed oritavancin MIC_50_/MIC_90_ values of 0.03 to 0.06/0.12 to 0.25 mg/L and susceptibility rates of 97.1% to 100.0%. The activity of oritavancin and comparator agents against Viridans group Streptococcus and beta-hemolytic Streptococcus are displayed in the supplemental material (Table S1).

In conclusion, we noted encouraging decreasing trends in MRSA and VRE rates. Oritavancin showed potent and consistent activity against Gram-positive pathogens that cause BSI in US from 2010 to 2019, including multidrug-resistant pathogens such as MRSA, MRCoNS, VRE, and E. faecium with elevated daptomycin MIC and reduced susceptibility to linezolid and daptomycin. Further studies are warranted to identify appropriate oritavancin dosing strategies and the role of oritavancin in the armamentarium against either susceptible or multidrug-resistant Gram-positive isolates causing BSI and other severe infections.
