# Supplementary material for: Activity of Oritavancin against Gram-Positive Pathogens Causing Bloodstream Infections in the United States over 10 Years: Focus on Drug-Resistant Enterococcal Subsets (2010–2019)
Source: Antimicrob Agents Chemother. 2022 Feb 15;66(2):e01667-21. doi: 10.1128/AAC.01667-21 (PMC8846398; doi:10.1128/AAC.01667-21)
Supplement: Supplemental file 1 — Supplemental tables. Download AAC.01667-21-s0001.pdf, PDF file, 0.4 MB [file aac.01667-21-s0001.pdf]

Table S1 Antimicrobial activity of oritavancin and comparator agents tested against *Staphylococcus spp. and Streptococcus spp.* isolates from bloodstream infections in US medical centers.

**Organism/Phenotype**	**MIC_{50}**	**MIC_{90}**	**MIC Range (mg/L)**	**CLSI [a]**	
				**%S[a]**	**%R[a]**
**MSSA (*n* = 4,272)**					
Oritavancin	0.03	0.06	≤0.008 to 0.25	99.8	-
Clindamycin	≤0.25	≤0.25	≤0.25 to >2	95.7	4.1
Daptomycin	0.25	0.5	≤0.12 to 4	>99.9	-
Erythromycin	≤0.25	>4	≤0.25 to >4	68.0	26.7
Levofloxacin	≤0.5	2	≤0.5 to >4	89.6	10.0
Linezolid	1	2	≤0.12 to 4	100.0	0.0
Minocycline	≤0.06	0.12	≤0.06 to >8	99.2	0.4
Oxacillin	0.5	0.5	≤0.25 to 2	100.0	0.0
Teicoplanin	≤2	≤2	≤2 to 4	100.0	0.0
Tetracycline	≤0.5	≤0.5	≤0.5 to >8	96.4	2.8
Trimethoprim-sulfamethoxazole	≤0.5	≤0.5	≤0.5 to >4	99.3	0.7
Vancomycin	1	1	≤0.12 to 2	100.0	0.0
**MRSA (*n* = 3,226)**					
Oritavancin	0.03	0.06	≤0.008 to 0.25	99.8	-
Clindamycin	≤0.25	>2	≤0.25 to >2	68.0	31.9
Daptomycin	0.25	0.5	≤0.12 to 2	99.8	-
Erythromycin	>4	>4	≤0.25 to >4	11.4	85.4
Levofloxacin	4	>4	≤0.5 to >4	27.2	71.4
Linezolid	1	1	≤0.12 to 4	100.0	0.0
Minocycline	≤0.06	0.25	≤0.06 to >8	98.0	1.0
Oxacillin	>2	>2	>2 to >2	0.0	100.0
Teicoplanin	≤2	≤2	≤2 to 8	100.0	0.0
Tetracycline	≤0.5	1	≤0.5 to >8	94.4	4.8
Trimethoprim-sulfamethoxazole	≤0.5	≤0.5	≤0.5 to >4	96.0	4.0
Vancomycin	1	1	≤0.12 to 2	100.0	0.0
**MS-CoNS (*n* = 709)**					
Oritavancin	0.03	0.06	≤0.008 to 1	-	-

Clindamycin	≤0.25	1	≤0.25 to >2	88.9	9.4
Daptomycin	0.25	0.5	≤0.12 to 2	99.9	-
Erythromycin	≤0.25	>4	≤0.25 to >4	56.1	40.9
Levofloxacin	≤0.5	4	≤0.5 to >4	82.8	15.9
Linezolid	0.5	1	≤0.12 to >8	99.9	0.1
Oxacillin	≤0.25	0.5	≤0.25 to 2	100.0	0.0
Teicoplanin	≤2	4	≤2 to 16	99.4	0.0
Tetracycline	≤0.5	8	≤0.5 to >8	89.8	8.3
Trimethoprim-sulfamethoxazole	≤0.5	4	≤0.5 to >4	87.3	12.7
Vancomycin	1	2	≤0.12 to 2	100.0	0.0
**MR-CoNS (*n* = 1,163)**					
Oritavancin	0.03	0.12	≤0.008 to 0.5	-	-
Clindamycin	0.5	>2	≤0.25 to >2	50.8	46.8
Daptomycin	0.25	0.5	≤0.12 to 1	100.0	-
Erythromycin	>4	>4	≤0.25 to >4	20.3	76.2
Levofloxacin	>4	>4	≤0.5 to >4	28.2	69.9
Linezolid	0.5	1	≤0.12 to >8	96.5	3.5
Oxacillin	>2	>2	1 to >2	0.0	100.0
Teicoplanin	≤2	8	≤2 to >16	97.6	0.2
Tetracycline	1	>8	≤0.5 to >8	80.4	19.2
Trimethoprim-sulfamethoxazole	2	>4	≤0.5 to >4	50.9	49.1
Vancomycin	1	2	≤0.12 to 4	100.0	0.0
**VGS (*n* = 921)**					
Oritavancin	0.015	0.12	≤0.008 to 0.5	98.7	-
Clindamycin	≤0.25	>2	≤0.25 to >2	86.2	12.3
Daptomycin	0.25	0.5	≤0.06 to >1	99.9	
Erythromycin	1	>4	≤0.25 to >4	42.0	55.5
Levofloxacin	1	>4	≤0.5 to >4	86.5	12.6
Linezolid	1	1	≤0.12 to 4	99.9	-
Penicillin	≤0.06	1	≤0.06 to >4	67.5	5.0
Teicoplanin	≤2	≤2	≤2 to ≤2	-	-

Tetracycline	≤0.5	>4	≤0.5 to >4	64.4	32.2
Trimethoprim-sulfamethoxazole	≤0.5	4	≤0.5 to >4	-	-
Vancomycin	0.5	0.5	≤0.12 to 1	100.0	-
**BHS (*n* = 1,394)**					
Oritavancin	0.03	0.25	≤0.008 to 1	98.5	-
Clindamycin	≤0.25	>2	≤0.25 to >2	79.4	19.9
Daptomycin	0.12	0.25	≤0.06 to 1	100.0	-
Erythromycin	≤0.25	>4	≤0.25 to >4	60.9	38.1
Levofloxacin	≤0.5	1	≤0.5 to >4	99.1	0.7
Linezolid	1	1	≤0.12 to 2	100.0	-
Penicillin	≤0.06	≤0.06	≤0.06 to 0.12	100.0	-
Teicoplanin	≤2	≤2	≤2 to ≤2	-	-
Tetracycline	>4	>4	≤0.5 to >4	42.4	55.9
Trimethoprim-sulfamethoxazole	≤0.5	≤0.5	≤0.5 to >4	-	-
Vancomycin	0.5	0.5	0.12 to 1	100.0	-

MRSA, methicillin-resistant *Staphylococcus aureus*; MSSA, methicillin-susceptible *S. aureus*; MR-CoNS, methicillin-resistant coagulase-negative *Staphylococcus*; MS-CoNS, methicillin-susceptible coagulase-negative *Staphylococcus*; VGS, Viridans group streptococci, BHS, β-hemolytic streptococci.

[a] Criteria as published by CLSI [1]

Table S2 Antimicrobial activity of oritavancin and comparator agents tested against *Enterococcus* isolates and resistant subsets from bloodstream infections in US medical centers.

**Organism/Phenotype** / **Antimicrobial agent**	MIC_{50}	MIC_{90}	**MIC Range (mg/L)**	**CLSI** %S[a]	**CLSI** %R[a]
Enterococcus faecalis					
**All (*n* = 1,709)**					
Oritavancin	0.015	0.06	≤0.008 to 0.5	97.5 [b]	
Ampicillin	≤1	2	≤1 to 4	100.0	0.0
Daptomycin	1	1	≤0.25 to 4	99.5	0.0
Levofloxacin [d]	1	>4	≤0.5 to >4	72.4	27.2
Linezolid	1	2	≤0.25 to >8	99.9	0.1
Teicoplanin	≤2	≤2	≤2 to >8	96.9	2.3
Tetracycline	>8	>8	≤1 to >8	25.2	74.2
Vancomycin	1	2	≤0.5 to >16	96.4	3.5
**Vancomycin NS (*n* = 62)**					
Oritavancin	0.25	0.5	0.008 to 0.5	40.3 [b]	
Ampicillin	≤1	2	≤1 to 2	100.0	0.0
Daptomycin	0.5	1	≤0.25 to 2	100.0	0.0
Levofloxacin [d]	>4	>4	≤0.5 to >4	8.1	91.9
Linezolid	1	1	0.5 to 2	100.0	0.0
Teicoplanin	>8	>8	≤2 to >8	14.5	62.9
Tetracycline	>8	>8	≤1 to >8	13.1	83.6
Vancomycin	>16	>16	8 to >16	0.0	96.8
**VanA phenotype (*n* = 53)**					
Oritavancin	0.25	0.5	0.015 to 0.5	32.1 [b]	
Ampicillin	≤1	2	≤1 to 2	100.0	0.0
Daptomycin	0.5	1	≤0.25 to 2	100.0	0.0
Levofloxacin [d]	>4	>4	1 to >4	7.5	92.5
Linezolid	1	1	0.5 to 2	100.0	0.0
Teicoplanin	>8	>8	4 to >8	0.0	73.6
Tetracycline	>8	>8	≤0.25 to >8	9.6	86.5
Vancomycin	>16	>16	>16 to >16	0.0	100.0

**VanB phenotype (*n* = 9)**					
Oritavancin	0.015		0.008 to 0.015	88.9 [b]	
Ampicillin	2		≤1 to 2	100.0	0.0
Daptomycin	0.5		0.5 to 1	100.0	0.0
Levofloxacin [d]	>4		≤0.5 to >4	11.	88.9
Linezolid	1		0.5 to 1	100.0	0.0
Teicoplanin	≤2		≤2 to ≤2	100.0	0.0
Tetracycline	>8		≤1 to >8	33.3	66.7
Vancomycin	>16		8 to >16	0.0	77.8
**Daptomycin-NS (MIC ≥4 mg/liter; *n* = 8)**					
Oritavancin	0.03		0.008 to 0.06	100.0 [b]	
Ampicillin	≤1		≤1 to 2	100.0	0.0
Daptomycin	4		4 to 4	0.0	0.0
Levofloxacin [d]	1		1 to >4	75.0	25.0
Linezolid	1		0.5 to 1	100.0	0.0
Teicoplanin	≤2		≤2 to ≤2	100.0	0.0
Tetracycline	>8		≤1 to >8	12.5	87.5
Vancomycin	1		1 to 2	100.0	0.0
Enterococcus faecium					
**All (*n* = 1,082)**					
Oritavancin	0.03	0.06	≤0.008 to 0.5	98.4 [b]	
Ampicillin	>8	>8	≤1 to >8	12.7	87.3
Daptomycin	2	2	≤0.25 to >8	99.2 [c]	0.8
Levofloxacin [d]	>4	>4	≤0.5 to >4	9.5	87.0
Linezolid	1	2	≤0.25 to >8	98.8	0.5
Teicoplanin	>8	>8	≤2 to >8	30.2	46.2
Tetracycline	>8	>8	≤1 to >8	23.9	74.1
Vancomycin	>16	>16	≤0.5 to >16	27.5	72.3
**Vancomycin NS (*n* = 784)**					
Oritavancin	0.03	0.12	≤0.008 to 0.5	97.8 [b]	
Ampicillin	>8	>8	≤0.5 to >8	0.6	99.4

Daptomycin	1	2	≤0.25 to >8	99.4 [c]	0.6
Levofloxacin [d]	>4	>4	4 to >4	0.0	99.5
Linezolid	1	2	≤0.25 to >8	98.9	0.4
Teicoplanin	>8	>8	≤2 to >8	3.7	63.8
Tetracycline	>8	>8	≤1 to >8	17.6	79.8
Vancomycin	>16	>16	8 to >16	0.0	99.7
**VanA phenotype (*n* = 755)**					
Oritavancin	0.03	0.12	≤0.008 to 0.5	97.7 [b]	
Ampicillin	>8	>8	≤0.5 to >8	0.4	99.6
Daptomycin	1	2	≤0.25 to >8	99.5 [c]	0.5
Levofloxacin [d]	>4	>4	4 to >4	0.0	99.5
Linezolid	1	2	≤0.25 to >8	98.9	0.4
Teicoplanin	>8	>8	4 to >8	0.0	66.2
Tetracycline	>8	>8	≤1 to >8	17.5	79.8
Vancomycin	>16	>16	>16 to >16	0.0	100.0
**VanB phenotype (*n* = 29)**					
Oritavancin	≤0.008	0.03	≤0.008 to 0.06	100.0 [b]	
Ampicillin	>8	>8	2 to >8	6.9	93.1
Daptomycin	1	2	≤0.25 to 8	96.6 [c]	3.4
Levofloxacin [d]	>4	>4	>4 to >4	0.0	100.0
Linezolid	1	1	0.25 to 4	96.6	0.0
Teicoplanin	≤2	≤2	≤2 to 8	100.0	0.0
Tetracycline	>8	>8	≤1 to >8	20.7	79.3
Vancomycin	>16	>16	8 to >16	0.0	93.1
**Daptomycin MIC, 2-4 mg/liter (*n* = 540)**					
Oritavancin	0.03	0.12	≤0.008 to 0.5	97.8 [b]	
Ampicillin	>8	>8	≤1 to >8	13.3	86.7
Daptomycin	2	4	2 to 4	100.0 [c]	0.0
Levofloxacin [d]	>4	>4	≤0.5 to >4	10.6	85.4
Linezolid	1	2	0.25 to 8	98.5	0.7
Teicoplanin	>8	>8	≤2 to >8	31.3	41.7

Tetracycline	>8	>8	≤1 to >8	20.6	78.0
Vancomycin	>16	>16	≤0.5 to >16	29.8	70.0
**Daptomycin-R (MIC ≥8 mg/liter; *n* = 9)**					
Oritavancin	0.015		≤0.008 to 0.25	88.9 [b]	
Ampicillin	>8		>8 to >8	0.0	100.0
Daptomycin	8		8 to >8	[c]	100.0
Levofloxacin [d]	>4		>4 to >4	0.0	100.0
Linezolid	2		0.25 to 4	88.9	0.0
Teicoplanin	8		≤2 to >16	55.6	44.4
Tetracycline	>8		>8 to >8	0.0	100.0
Vancomycin	>16		≤0.5 to >16	44.4	55.6
**Linezolid-NS (≥4 mg/liter; *n* = 13)**					
Oritavancin	0.015	0.06	≤0.008 to 0.12	100.0 [b]	
Ampicillin	>8	>8	>8 to >8	0.0	100.0
Daptomycin	2	4	1 to >8	92.3 [c]	7.7
Levofloxacin [d]	>4	>4	4 to >4	0.0	84.6
Linezolid	4	8	4 to >8	0.0	38.5
Teicoplanin	>8	>8	≤1 to >8	38.5	38.5
Tetracycline	>8	>8	≤0.25 to >8	7.7	92.3
Vancomycin	>16	>16	1 to >16	30.8	61.5
**Ampicillin-R (≥16 mg/liter; *n* = 945)**					
Oritavancin	0.03	0.12	≤0.008 to 0.5	98.2 [b]	
Ampicillin	>8	>8	>8 to >8	0.0	100.0
Daptomycin	2	2	≤0.25 to >8	99.0 [c]	1.0
Levofloxacin [d]	>4	>4	1 to >4	1.0	98.2
Linezolid	1	2	≤0.25 to >8	98.6	0.5
Teicoplanin	>8	>8	≤2 to >8	20.4	52.6
Tetracycline	>8	>8	≤1 to >8	17.6	80.1
Vancomycin	>16	>16	≤0.5 to >16	17.6	82.2

R, resistant; NS, non-susceptible.
[a] Criteria as published by CLSI [1]

[b] Using CLSI breakpoint approved for vancomycin-susceptible *E. faecalis* isolates.
[c] *E.faecium* susceptible based on a dosage regimen of 8-12 mg/kg [1].
[d] Uncomplicated UTI only.

REFERENCES

1. **CLSI.** 2021. M100Ed31. Performance standards for antimicrobial susceptibility testing: 31st informational supplement. Clinical and Laboratory Standards Institute, Wayne, PA.
